# Evaluating Digital Health Solutions in Diabetes and the Role of Patient-Reported Outcomes: Targeted Literature Review

**DOI:** 10.2196/52909

**Published:** 2025-06-04

**Authors:** Paco Cerletti, Michael Joubert, Nick Oliver, Saira Ghafur, Pasquale Varriale, Ophélie Wilczynski, Marlene Gyldmark

**Affiliations:** 1 Roche Diagnostics, Grenzacherstrasse 124, Basel, 4058, Switzerland, 41797072197; 2 Caen University Hospital - UNICAEN, Caen, France; 3 Department of Diabetes, Endocrinology, and Reproduction, Imperial College London, London, United Kingdom; 4 Carenity, Paris, France

**Keywords:** diabetes, patient-reported outcome, digital health solutions, people with diabetes, digital health, diabetic, diabetes mellitus, type 2 diabetes, type 1 diabetes, patient care, diabetic patients, health technology assessment, health technology, clinical trials, evaluation, treatment, health-related, diabetes care

## Abstract

**Background:**

Digital health solutions (DHS) are technologies with the potential to improve patient outcomes as well as change the way care is delivered. The value of DHS for people with diabetes is not well understood, nor is it clear how to quantify this value.

**Objective:**

We aimed to summarize current literature on the use of patient-reported outcome measures (PROMs) in diabetes as well as in selected guidelines for Health Technology Assessment (HTA) of DHS to highlight gaps, needs, and opportunities for the use of PROMs to evaluate DHS.

**Methods:**

We searched PubMed and ClinicalTrials.gov to establish which PROMs were most used in diabetes clinical trials and research between 1995 and May 2024. HTA guidelines on DHS evaluation from France, Germany, and the United Kingdom were also assessed to identify PROMs for DHS evaluation in general.

**Results:**

A total of 46 diabetes-specific PROMs and 16 nondiabetes-specific PROMs were identified. The most used diabetes-specific PROMs were (1) Diabetes Distress Scale, (2) Problem Areas in Diabetes, (3) Diabetes Empowerment Scale, (4) Diabetes Quality of Life, and (5) Diabetes Treatment Satisfaction Questionnaire. The most used nondiabetes-specific PROMs were Beck Depression Inventory, Sickness Impact Profile, EuroQol 5-Dimension, and Short Form 36-Item Health Survey. In HTA guidelines, the most prominent domain was health-related quality of life, for whose assessment there are well-established measures (Short Form 36-Item Health Survey and EuroQol 5-Dimension).

**Conclusions:**

Of the many PROMs used in diabetes care, few are currently used to evaluate DHS, and certain domains of value in diabetes are not mentioned in HTA guidelines. A common, comprehensive DHS-specific HTA framework could facilitate and accelerate the evaluation of DHS.

## Introduction

Despite innovative technologies and major advances in drug discovery, treatment goals in diabetes have not been fully met [1] and access for populations at risk is still lacking [2]. More holistic and integrated treatment modalities are needed to improve treatment goals and access to care for people with diabetes worldwide [3]. Digital Health Solutions (DHS) may offer a viable way to tackle these challenges. The potential of DHS for improving health and well-being is becoming increasingly evident [4], and health authorities have begun to acknowledge the benefits of these solutions for patients and health care systems [5-8].

The concept of digital health has been defined by the US Food and Drug Administration (FDA) as the use of technologies for health care and related purposes [9]. “Technologies” encompass mobile health, health information technology, wearable devices, telehealth and telemedicine, and personalized medicine, while “health care purposes” include preventing and treating disease, improving diagnosis, and enhancing health care delivery. The UK National Institute of Health and Care Excellence (NICE) characterizes digital health as technological solutions that improve (1) the efficiency of health systems, (2) understanding and communication about health, or (3) health interventions [5]. Digital health interventions provide health care stakeholders with a means to address unresolved health system challenges, according to the World Health Organization (WHO) [10].

Traditionally, outcomes of health interventions have been measured using “narrow” clinical, biological, and metabolic endpoints. However, the WHO’s definition of health is broad: “a state of complete physical, mental and social well-being and not merely the absence of disease or infirmity” [11]. By improving outcomes related to patients and health care systems, DHS may benefit patient’s health and well-being beyond clinically measurable values [4,9]. Assessing these patient-reported perceived elements, for example, empowerment, self-efficacy or health literacy, is central to holistically understanding the effects of health interventions and capturing the full range of benefits from DHS [12].

Compared with existing interventions, DHS could provide near-constant monitoring and feedback, helping patients to better understand their disease and supporting them in making health care decisions [10]. At a system level, DHS could automatically collect, manage, and store health data. By providing this information to health care professionals, the quality of care could improve, and encounters could become more effective. There is an emerging need to provide validated tools that reliably assess these parameters of health and health care benefits. Patient-reported outcome measures (PROMs) are expected to play a key role in understanding patients’ perspectives of the mentioned outcomes to fully understand the range of benefits from DHS.

PROMs are instruments to capture the impact of treatment on relevant patient perspectives and health-related outcomes usually outside the scope of clinical or biological endpoints. Examples include standardized, validated questionnaires on health status, perceived level of impairment, empowerment, or health-related quality of life PROMs used in health technology assessment (HTA) [6,7], which is defined by the WHO as “the systematic evaluation of properties, effects, and impacts of health-care technology” [13]. In this research, we will focus on the potential value of PROMs for assessing DHS for people with diabetes.

The objectives of this research are to (1) review current literature on the use of PROMs in diabetes and in selected HTA guidelines for DHS (2) and describe the challenges, needs, and opportunities for the use of PROMs to evaluate DHS.

## Methods

### Use of PROMs in Diabetes: Literature and HTA Guidelines Review

We conducted a literature review to identify the PROMs used in diabetes. In addition, 3 databases were scanned—ProQolid, ClinicalTrials.gov, and PubMed—as these are the main sources where research on PROMs is being published. ProQolid was used to identify diabetes- and nondiabetes-specific PROMs. Findings were complemented by data from literature reviews of PROs used in prominent diabetes outcome consortiums, such as the International Consortium for Health Outcomes Measurement. Furthermore, PubMed and ClinicalTrials.gov were searched to establish which PROMs were most used in diabetes clinical trials and research between 1995 and May 2024 to identify the use of these PROMs for evaluating DHS (MultimediaAppendices 1  2).

In PubMed, the PROs name and “diabetes” were used to search for relevant results and in ClinicalTrials.gov the PROs name and “type 1 diabetes” and “type 2 diabetes” were used. Then, to determine associations with DHS, PROs name and “diabetes” were used in conjunction with “mobile application,” “telemedicine,” “telehealth,” “health digital solutions,” and “e-health” in PubMed. Not all articles mentioning DHS were selected; 2 data scientists independently read all abstracts to identify the peer-reviewed publications where PROs were used in relation to DHS. Therefore, in the final list of selected publications, a DHS was used, as well as a PRO in relation to this DHS.

PROM type (diabetes-specific or nondiabetes-specific [generic]), domain and objective, and occurrences in literature were collected for each PROM. The number of items, format, and administration time were collected for disease-specific PROMs, and copyright and language versions were collected for generic PROMs. Any mentions of PROMs used in reimbursement studies were noted. The PROMs most used in diabetes were analyzed, by determining which PROMs appeared in all 3 PubMed and ClinicalTrials.gov searches and were mentioned in HTA guidelines, as described next.

Alongside this, we retrieved and assessed guidelines about DHS evaluation from health authorities in France (HAS), Germany (BfArM), and the UK (NICE) [5-8]. These countries were chosen because of recent initiatives by their HTA authorities offering guidance on DHS assessment (Textbox 1).

Textbox 1. **French, German, and UK health technology assessment guidelines for evaluating digital health solutions.**

Health authority Country (Year) and Guidelines

**Haute Autorité de Santé France [7] (2019)**

- Assessment of medical devices: Assessment principles established by the Medical Device and Health Technology Evaluation Committee (CNEDiMTS) to determine the reimbursement eligibility of medical devices for individual use

**Haute Autorité de Santé France [8] (2019)**

- 2019 Prospective Analysis Report - Digital technology: what (R)evolution? [in French]

**German Federal Institute for Drugs and Medical Devices (BfArM) Germany [6] (2020)**

- The Fast-Track Process for Digital Health Applications (DiGA) according to Section 139e SGB V - A Guide for Manufacturers, Service Providers and Users (version 1.0)

**National Institute of Health and Care Excellence UK [5] (2019)**

- Evidence standards framework for digital health technologies

## Results

Overall, 62 PROMs were identified from the literature review: 46 diabetes-specific PROMs and 16 nondiabetes-specific PROMs (Multimedia Appendix 3). The diabetes-specific PROMs most used in diabetes clinical trials and research are shown in Table 1.

**Table 1.** Most used diabetes-specific patient-reported outcome measures.

PROM^a^	Domain or objective	Items	Format	Admin time (min)	Occurrences					Mentions HTA^c^ guidelines or DHS^d^ reimbursement
					ClinicalTrials.gov^b^			PubMed		
					Diabetes, n	T1D^e^, n	T2D^f^, n	PROMs and diabetes^b^, n	PROMs and diabetes for digital solutions^g^, n	
Summary of Diabetes Self-Care Activities (SDSCA) [14]	Diabetes self-management	11	Self-report questionnaire with scores	<10	159	9	105	306	N/A9	^N/A^
Diabetes Distress Scale (DDS) [15]	Psychosocial distress	17	6-point Likert Scale	10	243	105	124	277	10	N/A
Areas in Diabetes (PAID) scale [16]	Emotional functioning	1, 5, 20	Questions with values from 0 (“no problem”) to 4 (“serious problem”)	NA	255	112	—	400	10	United Kingdom
Diabetes Empowerment Scale (DES) & DES-sf [17]	Diabetes-related psychosocial self-efficacy	8, 28	Response categories ranging from “strongly disagree” to “strongly agree”	NA	92	21	47	99	9	N/A
Diabetes Quality of Life (DQOL) [18]	Relative burden of an intensive diabetes treatment regimen	46	5-point Likert scale in 3 main domains: “satisfaction”, “impact”, and “worry”	NA	92	52	39	274	3	French^i^
Diabetes Treatment Satisfaction Questionnaire (DTSQ) [14]	Satisfaction with diabetes treatment regimens and changes in satisfaction with treatment	8	7-point scale ranging from 0 to 6. The questionnaire assesses treatment satisfaction and burden from hyper- and hypoglycemia	—	219	135	83	110	8	French^i^

a PROM: patient-reported outcome measurement.

b The keywords used were: “full name of PROM” AND diabetes; “full name of PROM” AND type 1 diabetes; “full name PROM” AND type 2 diabetes.

c HTA: health technology assessment.

d DHS: digital health solution.

e T1D: type 1 diabetes.

f T2D: type 2 diabetes.

g The keywords used were: “full name of PROM” AND diabetes AND (“mobile application” OR telemedecine OR telehealth OR “health digital solutions” OR “e-health”).

h not available.

i Haute Autorité de Santé. Assessment of medical devices: assessment principles established by the Medical Device and Health Technology Evaluation Committee (CNEDiMTS) to determine the reimbursement eligibility of medical devices for individual use (La Plaine Saint-Denis, France; 2019).

These PROMs cover a wide range of outcomes, including diabetes self-management (SDSCA [15]), psychosocial distress (DDS [16]) and diabetes distress (PAID [17]), diabetes-related psychosocial self-efficacy (DES [18]), relative burden of an intensive diabetes treatment regimen (DQOL [19]), and satisfaction with diabetes treatment (DTSQ [14]). Furthermore, 3 PROMs were mentioned in HTA guidelines: PAID [17] in UK guidelines [5], and DQOL [19] and DTSQ [14] in French guidelines [7].

Table 2 shows the most prominent nondiabetes-specific PROMs used in diabetes clinical trials and research. The domains covered by these PROMs include severity of depression (BDI [20]), patient dysfunction assessed through everyday behavior (SIP [21]), health outcome from interventions on a common scale (EQ-5D [22]), and generic health concepts (SF-36 [23]). Two PROMs were mentioned in 3 HTA guidelines: EQ-5D [22] in French guidelines [7,8], and SF-36 [23] in French and German guidelines [6,7].

**Table 2.** Most used nondiabetes–specific patient-reported outcomes measures.

PROM^a^	Domain or objective	Copyright	Language	Occurrences					Mentions
				ClinicalTrials.gov^b^			PubMed		
				Diabetes	T1D^c^	T2D^d^	PROMs and diabetes^b^	PROMs and diabetes for digital solutions^e^	HTA^f^ guidelines or DHS^g^ reimbursement
Beck Depression Inventory (BDI) [20]	Severity of depression in adults and adolescents	Aaron T. Beck	English +73 others	82	13	37	581	3	—^h^N/A
Sickness Impact Profile (SIP) [21]	Patient dysfunction measured via everyday behavior, generally related to disease	Johns Hopkins University, 1977	English	2	1	1	397	2	—N/A
EQ-5D [22]	Health outcome from interventions on a common scale, for evaluation, allocation, and monitoring	EuroQoL Group	English +181 others	312	47	157	895	8	French^i^ United States^j^ (specific example, FDA)
Short Form (SF-36) [23]	Generic health concepts relevant across age, disease, and treatment groups	Medical Outcomes Trust (MOT), Dr J. Ware	English +160 others	392	39	195	1387	10	French German^k^ (specific example) United States (specific example, FDA)

a PROM: patient-reported outcome measure.

b The keywords used were: “full name of PROM” AND diabetes; “full name of PROM” AND type 1 diabetes; “full name PROM” AND type 2 diabetes.

c T1D: type 1 diabetes.

d T2D: type 2 diabetes.

e The keywords used were: “full name of PROM” AND diabetes AND (“mobile application” OR telemedecine OR telehealth OR “health digital solutions” OR “e-health”)

f HTA: health technology assessment

g DHS: digital health service.

h Not available.

i Haute Autorité de Santé. Assessment of medical devices: assessment principles established by the Medical Device and Health Technology Evaluation Committee (CNEDiMTS) to determine the reimbursement eligibility of medical devices for individual use (La Plaine Saint-Denis, France; 2019).

j Food & Drug Administration Center for Devices and Radiological Health. Value and Use of Patient-Reported Outcomes (PROs) in Assessing Effects of Medical Devices - CDRH Strategic Priorities 2016-2017. (Silver Spring, MD; 2017).

k Federal Institute for Drugs and Medical Devices (BfArM). The Fast-Track Process for Digital Health Applications (DiGA) according to Section 139e SGB V - A Guide for Manufacturers, Service Providers and Users (version 1.0) (Bonn, Germany; 2020).

The review of the 3 HTA guidelines identified 12 recommended patient outcomes and 5 outcome categories (Table 3). The most prominent domain, Quality of Life, was recommended by the 3 HTA guidelines and there were well-established nondiabetes-specific PROMs for its assessment, for example, SF-36 and EQ-5D [22,23]. EQ-5D is directly linked to reimbursement processes in many countries and is used to derive quality-adjusted life year. The next most prominent domain was acceptability, from the DHS outcome category. Acceptability was highly relevant in both French and UK HTA guidelines [5,7]. Two diabetes-specific PROMs were mentioned in French HTA guidelines: DTSQ [14] for the assessment of acceptability, and DQOL [19] for quality of life (QOL) assessment.

**Table 3.** Domains identified in French, UK, and German health technology assessment guidelines for evaluating digital health solutions. +: outcome mentioned; ++: special attention paid to this outcome; +++: outcome of great importance (patient-reported outcomes measures in guideline linked to outcome, if mentioned).

Outcome category		France (HAS^a^)	United Kingdom (NICE^b^)	Germany (BfArM)^c^
		Assessment of medical devices: assessment principles established by the CNEDiMTS^d^ [7]	Evidence standards framework for digital health technologies^e^ [5]	The Fast-Track process for digital health applications (DiGA)^f^^,^^g^ [6]
Acceptability				
DHS^h^		++ (DTSQ^i^ [14])	++^j^	
User satisfaction^k^				
DHS			+^l^	+
Engagement				
DHS			+^l^	
Patient empowerment^k^				
Patient sovereignty			++^l^	
Health literacy				
Patient sovereignty				+ (HLS-EU-Q^m^ [24])
Quality of life^k^				
^n^QoL/disease management		+++ (EQ-5D [22], SF-36 [23], DQOL^o^ [19])	+++^l^	+++ (SF-36^p^ [23])
Symptom severity				
QoL/disease management			+^l^	+++ (NRS^q^ [25], SCL-90^r^ [GSI^s^, PSDI^t^, PST]^u^ [26])
Autonomy				
QoL/disease management		+	+^l^	+
Coping with illness-related difficulties				
QoL/disease management				+
Reduction of therapy-related effort and strain (for patients or relatives)				
QoL/disease management		+		+
Adherence				
Adherence				++ (MAQ^v^ [27], Morisky Score [28])
Enhanced safety				
Safety				+

a HAS: Haute Autorité de Santé.

b NICE: National Institute for Health and Care Excellence.

c BfArM: [German] Federal Institute for Drugs and Medical Devices.

d CNEDiMTS: [French] Medical Device and Health Technology Evaluation Committee.

e DHT: digital health technology.

f All PROMs mentioned were given in specific examples.

g DiGA: digital health application.

h DHS: digital health solution.

i DTSQ: Diabetes Treatment Satisfaction Questionnaire.

j Applies to DHTs in tiers 1, 2, 3a and 3b.

k Domain covered satisfactorily by existing diabetes-specific PROMs.

l Applies to DHTs in tiers 3a (disease prevention and management) and 3b (treatment, diagnostic, active monitoring, and calculation tools with measurable user benefits) only.

m HLS-EU-Q: European Health Literacy Survey Questionnaire.

n QoL: quality of life.

o DQOL: Diabetes Quality of Life [instrument].

p SF-36: Short Form (36) Health Survey.

q NRS: Numerating Rating Scale.

r SCL-90: Symptom Checklist 90.

s GSI: Global Severity Index.

t PSDI: Positive Symptom Distress Index.

u PST: Positive Symptom Total.

v MAQ: Medication Adherence Questionnaire.

## Discussion

### Principal Findings

In this targeted literature review, we identified 46 diabetes-specific and 16 nondiabetes-specific PROMs that were most used in diabetes clinical trials and research between 1995 and May 2024. In addition, this review shows that HTA guidelines on DHS evaluation from France, Germany, and the United Kingdom primarily reflect well-established PROMs for health-related quality of life.

### Challenges in Using PROMs for the Evaluation of DHS

Our review highlights that major HTA bodies acknowledge the importance and emerging needs of accepting PROMs as valuable outcomes to evaluate digital health interventions. However, by comparing the patient outcomes recommended in these guidelines to the number of prominent PROMs listed in the literature, there is still a lot of room for increasing the adoption and recommendation of PROMs by HTA bodies. Overall, in the literature as well as in the guidelines we identified a gap for PROMs that are relevant in evaluating DHS, such as disease knowledge (eg, carb counting in diabetes), eHealth literacy, digital burden (eg, data overload, fear of digital surveillance, and adverse effects associated with using digital technology), sexual life, family life, or well-being at work.

PROMs that were developed decades ago, as many of the PROMs we found in the literature, are likely to require updating before they can be used to assess the value of DHS. This is because they were developed to capture the impact of other types of interventions, and they may not be relevant for DHS and how these might affect patients’ overall well-being. In addition, more research needs to be done into associations between the domains measured by the PROMs and other outcomes, for example, clinical and economic outcomes that are important in a reimbursement process.

Although we see PROMs being used more frequently in modern diabetes interventions [29], their use is currently neither widespread nor consistent. One of the reasons that few DHS-specific PROMs have been used until now is the relative “youth” of digital health. The term “digital health” was coined in 2000 [30], and the WHO published a guide to harmonize the use of digital health terminology as recently as 2018. By contrast, diabetes-specific PROMs first appeared in the late 1980 s—Self-Efficacy for Diabetes (SED-D) [31] and the Hypoglycemic Fear Survey (HFS) [32] appeared in 1987—while generic, nondiabetes-specific PROMs used in diabetes first appeared even longer ago in the 1960 s, for example, the Beck Depression Inventory (BDI) [20] in 1961 and the Affect Balance Scale (ABS) [33] in 1969. The difference in PROMs and development lifecycles of digital products means that DHS may require existing PROMs to be adapted to a digital environment [34] or for new, bespoke PROMs to be created to adequately characterize the value and impact of DHS on patients’ health perceptions, QOL, and general well-being [35]. Either way, the lack of PROMs related to specific attributes of DHS needs to be addressed.

Specific, validated PROMs related to diabetes DHS have already been developed [36,37]. For new PROMs, patient communities emphasize the importance of assessing outcomes such as numeracy or health literacy, coping with diabetes, knowledge in diabetes management, evaluating problems with DHS (eg data overload), and evaluation of trust in health care providers.

The HTA bodies of France, Germany, and the UK generally agree that PROMs are valuable for the evaluation of DHS, but the scope of current HTA guidelines is limited to specific types of DHS, eg, medical devices or digital health applications. For these HTA bodies, there is broad consensus about the importance of assessing DHS based on functional, technical, and organizational characteristics, such as data security, practicality, quality, interoperability, and safety. Moreover, not only the investigated HTA bodies and countries focus on reimbursing and evaluating DHS, but most developed countries are working on frameworks and policies to provide faster access to DHS for patients and people with diabetes [38]. Obtaining a consensus on the best way to use PROMs to evaluate DHS is a necessary next step.

In France, although different types of DHS have been approved for reimbursement (mobile apps, telemonitoring systems, etc), the principles of evaluation published by the French health authority are specific to medical devices [7]. Deliberation about future frameworks for digital health interventions (including medical devices with artificial intelligence) is currently ongoing at the request of the French government and pharmaceutical companies. In the guideline specific to medical devices, PROs are highly recommended for supporting claims for reimbursement. Specific PROs are listed to demonstrate the impact on QOL [7].

In 2019, Germany adopted the Digital Health care Act (DVG), to promote the use of telehealth, mobile apps, and other digital solutions, as well as the use of health data for research purposes. The DVG entitles all individuals covered by statutory health insurance to reimbursement for certain digital health applications. The manufacturer must provide evidence of the positive effect of the digital application on care. In the Fast Track process guideline [6], which details how mobile app manufacturers can apply for reimbursement, the BfArM defines a full set of requirements for DiGAV (digital health applications), such as the types of study expected, and provides examples of PROs that would be suitable for the evaluation of certain endpoints. However, the scope of the DVG is limited to lower-risk medical devices, and many potentially valuable digital health applications are therefore not covered by the provisions of the DVG.

Early in 2019, NICE published the Evidence Standards Framework for Digital Health Technologies [5]. It is not suitable for all digital solutions, as it excludes mobile applications directly downloaded on app stores by users and solutions incorporating artificial intelligence using adaptive algorithms. In this guideline, digital solutions are classified in 4 categories or tiers: the higher the category, the more important the request for evidence is. Endpoints may vary depending on the category. Using PROs is highly recommended.

### Needs and Opportunities of Using Patient-Reported Outcome Measures for the Evaluation of DHS

A major need in diabetes care is to improve behavior change techniques. Existing evidence shows that behavioral change can improve disease trajectories and reduce the risk of severe complications [39]. PROMs are at the core of understanding patient-relevant endpoints and how these relate to behavioral change as well as helping us understand where the unmet needs for people with diabetes are. By systematically assessing these outcome domains, therapy and behavioral decisions can be personalized for people with diabetes and therefore maximize the benefit of diabetes treatment. DHS may be a key catalyst for improving adherence to behavior change techniques and treatment overall [40]. By comprehensively understanding the effects of digital health interventions on both metabolic and patient-reported outcomes, people with diabetes may feel better informed about and included in treatment decisions, which has been shown to positively affect adherence and long-term outcomes [41].

As technologies quickly develop and become more available, it is vital to identify the unmet needs, through PROs, to properly assess the value of these solutions. As touched upon before, personalization of treatment plays a major role in increasing quality-of-care standards. The identification of unmet needs in outcome domains such as patient empowerment, QOL or health literacy should be a major deciding factor when tailoring treatment to people with diabetes. Much research is still needed in this area to provide evidence for unmet needs and PROMs tailored for DHS.

### The Need for a Holistic Treatment Approach

A holistic approach to improving health in diabetes looks beyond the attainment of established clinical endpoints and aims at including patient relevant dimensions. For example, improving metabolic control by reducing HbA1c can enhance QOL in diabetes, but it is not the only way of doing so. Decreased physical functioning and well-being of people with diabetes leads to a diminished QOL, but people with diabetes can actively improve QOL themselves through empowerment and self-efficacy [42,43]. Empowerment is the process by which patients gain the information and confidence to make independent, educated decisions about their diabetes using solid reasoning skills. Empowerment helps patients feel involved in treatment decisions and supports the ideal doctor-patient relationship, as defined in this quote, “The doctor is there to give the patient all the information the patient needs in order that the patient can make a decision, and the doctor should then implement the decision once the patient has made it” [44]. Self-efficacy is a measure of patients’ ability to manage their own diet, exercise, and medical treatment with assurance. DHS offer people with diabetes a way to increase empowerment and self-efficacy and thus both QOL and clinical outcomes. The number of DHS available to people with diabetes is increasing [45,46], which bodes well for patient-driven approaches to improving QOL and clinical outcomes in diabetes.

### Harmonizing the Evaluation of DHS

A common, comprehensive HTA framework to guide the use of PROMs in the context of DHS would help harmonize the evaluation of DHS. This, in turn, would facilitate and expedite the path of DHS to market and ultimately enhance the quality of support and education for people with diabetes. At present, HTA guidelines differ in terms of scope, types of DHS evaluated, and the role PROMs play in DHS reimbursement and evaluation [5-7]. These differences make the evaluation of DHS more complex and slower than it needs to be. Difficulties with evaluation may partly explain why DHS are struggling to be accepted by health systems despite promising results, and the harmonization of outcome measures may be one critical stepping-stone to simplify and accelerate the evaluation of DHS [47].

### Limitations of the Targeted Literature Review

While interpreting the results of this targeted literature review, the following limitations need to be considered. First, it is difficult to describe DHS as one category as they vary substantially in terms of disease area, treatment regimen, type, and their regulatory risk classification. Second, as this review reflects on the two databases PubMed and Clinical Trial.gov trials that are listed in other databases may not be captured. Third, the patients’ perspective is missing in this research although it is a key topic around the use of PROMs for evaluating DHS. Finally, our assessment focused only on HTA-driven European countries.

### The Impact and Challenges of PRO Data Collection

A crucial prerequisite for the use of PROMs is the cooperation of patients in data collection. Patients may not be willing to share this data, for example for data privacy reasons or because the respective PROMs are too tedious to fill in. For example, Skovlund et al [48] described that patients ask for shorter questionnaires with dichotomous items instead of Likert-scale response formats to minimize complexity, to lower the cognitive burden, and to reduce the risk of questionnaire fatigue. Moreover, it is important that patients’ data is collected accurately and the entry of wrong data is avoided. Therefore, the items’ wording and terminology needs to be clear to prevent misleading or misinterpretation [48]. Ultimately, the data collection method can also influence the use and the validity of PROMs as there might be a difference between data collected by digital tools (eg smartphone apps and SMS text messaging) used by the patients on their own versus paper-based questionnaires filled in at the doctor’s office.

### Conclusions

Many PROMs are used in diabetes care, although few currently exist with the aim to evaluate DHS. Certain domains of value to people with diabetes have few or no PROMs to evaluate them at present. Generally, key HTA bodies are acknowledging the value of PROMs, but there is a need to harmonize the outcomes and evaluation processes for DHS between countries. In diabetes, PROMs can help provide a more holistic assessment of patient health beyond the control of clinical and metabolic outcomes. Therefore, the value of DHS may be best captured through PROMs, which will increase our understanding of the full range of benefits from these interventions.

## Supplementary material

10.2196/52909 Multimedia Appendix 1 List of all assessed PROMs.

10.2196/52909 Multimedia Appendix 2 Search strategy per database.

10.2196/52909 Multimedia Appendix 3 PROMs, assessed HTA guidelines and expert opinions.

## Abbreviations

ABS: Affect Balance Scale
BDI: Beck Depression Inventory
CNEDiMTS: Medical Device and Health Technology Evaluation Committee
DHA: Digital Health care Act
DHS: digital health solutions
FAD: Food and Drug Administration
HFS: Hypoglycemic Fear Survey
HTA: health technology assessment
MOT: Medical Outcomes Trust
NICE: National Institute of Health and Care Excellence
PROM: patient-reported outcomes measure
QOL: quality of life
SED-D: Self-Efficacy for Diabetes
SIP: Sickness impact profile
WHO: World Health Organization
