# Supplementary material for: Evaluating Digital Health Solutions in Diabetes and the Role of Patient-Reported Outcomes: Targeted Literature Review
Source: JMIR Diabetes. 2025 Jun 4;10:e52909. doi: 10.2196/52909 (PMC12158397; doi:10.2196/52909)
Supplement: Multimedia Appendix 1 [file diabetes-v10-e52909-s001.pdf]

PROs (diabetes specific)	Year / Copyright	Reference article	Domain and/or Objective	Languages	Clinicaltrial.gov (Occurrences) criteria: "full name PRO" diabetes "full name PRO" diabetes type 1 "full name PRO" diabetes type 2	Pubmed (Occurrences) criteria: "full name PRO" diabetes "full name PRO" diabetes type 1 "full name PRO" diabetes type 2	Pubmed (Occurrences) criteria: "full name PRO" AND Diabetes and ("Mobile application" OR Telemedecine OR telehealth OR "health digital solutions" OR "e-health")	Description (only for the 5 most used PROs based on the number of occurrences of the columns 'F-G-H', taking into account colomn O as well)	References articles (only for the 5 most used PROs based on the number of occurrences of the columns 'F-G-H', taking into account colomn O as well)
**Adherence**									
**Adherence in Diabetes Questionnaire Insulin pump (ADQ-I)<br>Adherence in Diabetes Questionnaire conventional treatment (ADQ-C)**	**2012 /American Diabetes Association.**	Kristensen - 2012 - Psychometric Evaluation of the Adherence in Diabetes Questionnaire (ADQ-I-C)	Adherence behavior of children and adolescents TD1 and their caregivers	**Danish, English**	NA	NA	NA	-	-
**Adherence to Refills and Medications Scale for Diabetes - ARMS-D**	**2013 / (No information for copyright)**	Mayberry - 2013 - The ARMS-D out performs the SDSCA, but both are reliable, valid, and predict glycemic control	To assess the adherence to the treatment<br><br>The ARMS-D yields a total score plus two sub-scale scores (refill sub-scale and medication taking sub-scale).	**English + 11 more**	18	1	NA	-	-
**Self-Management**									
**Summary of Diabetes Self-Care Activities (SDSCA)**	**2000 / Oregon Research Institute**	Toobert et al., - 2000 - The Summary of Diabetes Self-Care (SDSCA)	is a brief self-report questionnaire of diabetes self-management that includes items assessing the following aspects of the diabetes regimen:<br>general diet, specific diet, exercise, blood-glucose testing, foot care, and smoking **DURING the PAST 7 DAYS.**	**English + 20 other**	105 (5 TD1, 65 TD2)	**202**	6	The original SDSCA measure (7) assessed 5 aspects of the diabetes regimen:general diet, specific diet, exercise, medication<br>taking, and blood-glucose testing. More recent studies using the scale have also included items on foot care and smoking. Similar to the Rand Corporation questionnaire, respondents report on the frequency with which they performed various activities over the previous 7 days. The SDSCA assesses levels of self-care and not adherence or compliance to a prescribed regimen<br>because of the difficulties associated with identifying, for a given patient, a specific unchanging standard against which behavior should be compared (10,26,27). (from Toobert	To determine the effect of mobile learning (application) on self-care behaviors and blood glucose control of type 2 diabetic patients. Positive effect of the mobile application on self-care behavior **(Hooshmandja, 2020)**<br><br>Enhance patient activation and self-management of T2D using the US Department of Defense's Mobile Health Care Environment in a patient-centered medical home setting. Significant improvement in SDSCA. (Gimbel, 2020)<br><br>Determine if **BlueStar app** led to improvements in HbA1c, patient self-management, experience of care, and self-reported health utilization. No significant results. **(Agarwal,**
**Diabetes Self-Management Profile (DSMP)**	**2000 /** The Diabetes Research in Children Network (DirecNet) Study Group -<br>**without copyright**	Harris - 2000 - Validation of a Structured Interview for the Assessment od diabetes Self-Management	To measure self-management of type 1 diabetes in children<br><br>Five areas of diabetes self-management: Exercise; Hypoglycemia; Diet; Blood Glucose Testing; and Insulin.	**English**	9 (4 TD1, 3 TD2)	29	NA	-	-
**Diabetes Management Self-Efficacy Scale (DMSES)**	**1999 / No information**	Bijl - 1999 - The psychometric properties of the diabetes management self-efficacy scale for TD2	Self-management behavior<br><br>4 subscales: nutrition specific and weight, nutrition general and medical treatment, physical exercise, and blood sugar.	**English, Italian**	15 (NA, 10 TD2)	31	NA	-	-
**Perceived Competence for Diabetes Scale (PCDS)**	**1998 / No information**	Not found	Assesses the degree to which people with diabetes feel they can manage the every-day aspects of diabetes care	**English**	30 (10 TD1, 16 TD2)	1	NA	-	-
**Diabetes Self-Management Questionnaire (DSMQ) &<br>Diabetes Self-Management Questionnaire-Revised (DSMQ-R)**	**2013, 2015, Dr Andreas Schmitt**	Schmitt et al. - 2013 - DSMQ development and evaluation of an instrument to assess diabetes self-care activities	To assess diabetes self-care activities associated with glycaemic control (TD1, TD2)	**Original (German), English, Spanish**	17	27	NA	-	-
**Appraisal of Diabetes Scale (ADS)**	**1991 / Prof. Michael P Carey**	Carey - 1991 - Reliability and Validity of the Appraisal of Diabetes Scale	To assess a diabetic person's appraisal of his or her disease. In what extent patients can take care of their diabetes and the impact on patient's QoL	**English + 2 more**	2	6	NA	-	-
**Emotional and social impact**									
**Diabetes Distress Scale (DDS)**	**2005 / Behavioral Diabetes Institute**	Polonski - 2005 - Assessing Psychosocial Distress in Diabetes	To assess psychosocial distress in diabetes:<br>- emotional burden<br>- regimen distress<br>- interpersonal distress<br>- physician distress	**English + 34 other**	135 (53 TD1, 63 TD2)	**142**	5	The Diabetes Distress Scale (DDS) is a 17-item scale that was developed specifically to assess 4 domains (subscales) of distress associated with diabetes care: emotional burden (5 items), physician-related distress (4 items), regimen-related distress (5 items), and diabetes-related interpersonal distress (3 items). Each item is rated on a 6-point scale, ranging from 1 (not a problem) to 6 (a very serious problem). The final score reported is an average score of the 17 items. DDS scores can be defined into 3 categories: little or no distress (DDS <2.0), moderate distress (DDS = 2.0-2.9), and high distress (DDS ≥3.0).<br>Based on previous research studies, the measure demonstrates strong internal consistency (α > 0.87) and	To verify if telemonitoring can help improve glycemic control in type 2 diabetes and can support empowerment to self-manage diabetes. DDS score wer reduced at two different time points (until 3 months) when compared to baseline **(McGloin, 2020)**<br><br>To assess the impact of a home telemedicine clinic model (CoYoT1 Clinic) on psychosocial and behavioral outcomes designed for young adults (YAs) with type 1 diabetes (T1D). **(Bakhack, 2019)**<br><br>Significant reductions in total mean (SD) DDS scores from baseline to 6 months were observed. The reduction in
**Problem Areas in Diabetes scale (PAID)**	**1995 / Joslin Diabetes Center**	Polonsky - 1995 - Assessment of Diabetes-Related Distress	To assess emotional functioning in diabetes	**English + 12 other**	154 (59 TD1, 71 TD2)	**261** (86, 143)	4	The PAID survey is a 20-item measure that describes common problematic situations for individuals with type 1 or type 2 diabetes, each representing a unique area of diabetes-specific emotional distress.<br>distress to frustration with aspects of the diabetes regimen. On a 6- point Likert scale, patients rate the degree to which each item is currently problematic for them, with 1 being no problem to 6 being a serious problem. The higher the score, the more diabetes-related stress	To test if the Follow-up care provided via telemedicine (TM) is a more integrated care pathway to manage diabetes-related foot ulcers (DFU) than traditionally-delivered healthcare.<br>No differences were found between the two groups. **(Iversen, 2020)**<br><br>Used with other PROs, Summary of Diabetes Self-Care Activities (SDSCA) and EuroQol-5D (EQ-5D).<br>**In the study BlueStar app, first app in United States to be given Food and Drug Administration approval as a mobile prescription therapy was used. (Agarval, 2019)**
**DHP-1 (Diabetes Health Profile)<br><br>DHP-18 (eDHP-18) (adaptation of DHP-1)**	**1996 / DHP Research and Consultancy Ltd<br><br>2000 / DHP Research and Consultancy Ltd**	Meadows - 1996 - The Diabetes Health Profile (DHP) - A New instrument for assessing the psychological profile of insuline...<br><br>Meadows - 2000 - Adapataion od DHP-1 for use with TD2....	Psychological and behavioural impact that diabetes can have on a person's daily living<br><br>Developed for Type 2 - NIDDM and Type 2 - Insulin-requiring patients. Used for Type1 and Type 2 diabetes	**English + 15 other<br><br>English + 31 other**	1<br><br>4	8<br><br>13	NA	-	-
**Brief Diabetes Distress Screening Instrument (DDS2)**	**2008 / No information**	Fisher - 2008 - Development of a brief diabetes distress screening instrument	To assess diabetes-related emotional distress	**English**	3	1	NA	-	-
**Type 2 Diabetes Stigma Assessment Scale (DSAS-2)**	**2016 / The Australian Centre for Behavioural Research in Diabetes (ACBRD)**	Browne - 2016 - Measuring the Stigma Surrounding TD2: Development and Validation of the Type 2 Diabetes Stigma Assessment Scale (DSAS-2)	To measure perceived and experienced stigma related to living with type 2 diabetes	**English, Spanish for USA**	NA	1 (1 TD2)	NA	-	-
**Diabetes Symptom Checklist-Revised (DSC-R)<br><br>Diabetes Symptom Checklist-Revised (DSC-2) - used in the context of gestational diabetes**	**1994 / EMGO Instituut 1998.**	Grootenhuis - 1994 - Development of a Type 2 Diabetes Symptom Checklist - a measure of symptom severity	To measure both the occurrence and the perceived burden of physical and psychological symptoms related to type 2 diabetes and its possible complications	**German (original) + 48 more**	13 (DSC-R), 1(DSC-2)	20	NA	-	-
**Diabetes Care Profile (DCP)**	**1998 / The University of Michigan**	Fitzgerald JT - 1996 - Development and validation of the Diabetes Care Profile	To assess the social and psychological factors related to diabetes and its treatment	**English, Chinese, Spanish for Usa**	3	**36**	NA	-	-
**Acceptance**									

**Diabetes Acceptance Scale (DAS)**	**2014 / Andreas Schmitt**	Schmitt - 2018 - Measurement of psychological adjustment to diabetes with the diabetes acceptance scale	To measure diabetes acceptance (TD1 & TD2)	**Original (German), English**	75 (6 TD1, 49 TD2)	**90**	NA	-	-
**Diabetes Medication Satisfaction Tool (DMSAT)**	**2008 / No information**	Anderson et al. - 2008 - (DMSAT) Diabetes Medication Satisfaction Tool A focus on treatment regimens	Patient acceptability and satisfaction with the use of diabetes medication therapy in their daily life	**English**	NA	2	NA	-	-
**Quality of life in diabetes**									
**Diabetes Quality of Life (DQOL)**	**1998 / Public domain**	Jacobson - 1994 - The_Evaluation_of_Two_Measures_of_Quality_of_Life TD1 & TD2	To assess the relative burden of an intensive diabetes treatment regimen	**English + 5 other**	51 (33 TD1, 17 TD2)	**212**	NA	-	-
**Diabetes specific quality of life scale (DSQOLS)**	**1998 / Uwe Bott**	Bott U, Muhlhauser I, Overmann H, Berger M. Validation of a diabetes-specific quality-of-life scale for patients with type 1 diabetes. Diabetes Care 1998; 21:757-69	To assess individual treatment goals among patients with type 1 diabetes	**German**	5	19	1	-	-
**Audit Diabetes Dependant Quality of life (ADDQOL) - 13 items (The original)**<br>**Audit Diabetes Dependant Quality of life (ADDQOL) - 18 items**<br>**Audit Diabetes Dependant Quality of life (ADDQOL) - 19 items**	**1990 / Professor C Bradley**<br>**2002**<br>**2006**	Wee - 2006 - Usefulness of the Audit of od ADDQoL questionnaire in paitents with diabetes	To measure individuals' perceptions of the impact of diabetes on their quality of life	**English + 62 other**	47 (9 TD1, 33 TD2)	**112**	NA	-	-
**D-39 (Diabetes-39)**	**Difficult to have the exact information**	More article are available	To assess quality of life	**Many versions available (we need to research further)**	3	29	NA	-	-
**Diabetes Impact Measurement Scales (DIMS)**	**1992 / No Information**	Hammond - 1992 - Measurement of Health Status in Diabetic Patients	To measure health status in adult type I and type II diabetic patients	**English + 3 other**	21 (1 TD1, 15 TD2)	3	NA	-	-
**Diabetes Quality of Life Clinical Trial Questionnaire (DQLCTQ)**	**1999 / IQVIA**	Shen - 2009 - Development and Validation of the Diabetes Quality of Life Clinical Trial Questionnaire	To measure the quality of life of diabetic patients in clinical trials	**English + 47 other**	2	1	NA	-	-
**Pediatric Quality of Life Inventory™ 3.0 & 3.2 Diabete Module (PedsQL™)**	**1998 / JW Varni**	Varni JW - 2003 - The PedsQl in Type 1 adn Tupe 2 diabetes<br><br>Varni JW - 2018 - Pediatric Quality of Life Inventory (PedsQL) 3.2 Diabetes Module for youth with Type 2 diabetes-reliability and validity	Is a brief measure of health-related quality of life in children and young people. The measure can be completed by parents (the Proxy Report) as well as children and young people (the Self-Report).	**English**	59 (48 TD1)	**103**	NA	-	-
**Empowerment & health literacy**									
**Diabetes Empowerment Scale (DES) & DES-sf**	**2000-2003 / The University of Michigan**	Anderson - 2000 - The Diabetes Empowerment Scale	To assess diabetes-related psychosocial self-efficacy	**English, Chinese, Spanish**	70 (17 TD1, 34 TD2)	**79**	7	The questionnaire also included an 8-question instrument, the Diabetes Empowerment Scale-Short Form (DES-SF), developed and<br>validated in a group of 239 African American subjects by the Michigan Diabetes Research and Training Center (Figure 3) [17]. This instrument is graded on a score of 1 (low self-efficacy) to 5 (high self-efficacy) and allows for an assessment of patients' diabetes-related self-efficacy [17]. (From Goh, 2015)	Evaluation of the clinical effectiveness and feasibility and the patients' and health care providers' experiences of a 12-week telemonitoring intervention with telephone support for patients commencing insulin therapy, focusing on the impact on patient empowerment. A significant increase in the mean DES score occurred. **(McGloin, 2020)**<br><br>To evaluate the effectiveness of individual empowerment strategies in patients with diabetes mellitus, through a systematic review performed in the PubMed, Scopus, Science Direct and BVS. Almost half of the studies used the DES. This systematic review showed that individual strategies for DM empowerment were not effective in [illegible]
**Literacy assessment for Diabetes (LAD)**	**2001 / No information about copyright**	Nath CR - 2001 - Development and validation of a literacy assessment tool for persons with Diabetes	To measure a patient's ability to pronounce terms that they would encounter during clinic visits and in reading menu and self-care instructions.	**English**	11	4	NA	-	-
**Self-Efficacy for Diabetes (SED-D)**	**1987 / No information**	Grossman - 1987 - Self-Efficacy in Adolescent Girls and boys with Insulin-dependent Diabetes Mellitus	Self-efficacy	**NA**	NA	NA	NA	-	-
**Satisfaction with and perception about treatment**									
**Diabetes Treatment Satisfaction Questionnaire (DTSQ)**<br>**Diabetes Treatment Satisfaction Questionnaire, status version (DTSQs)**<br>**Diabetes Treatment Satisfaction Questionnaire, change version (DTSQc)**	**1990 / Clare Bradley c.bradley@rhul.ac.uk.**<br>**2007**	Bradley et al. - 2007 - (DTSQc) shows greater responsiveness to improvements than the original DTSQ	To measure satisfaction with diabetes treatment regimens in people with diabetes and changes in satisfaction with treatment	**English + 121 other**	127 (55 TD1, 76 TD2)<br>88 (28 TD1, 60 TD2)<br>49 (20 TD1, 32 TD2)	**41**	5	The DTSQ was designed to assess diabetes treatment satisfaction in specific areas and perceived frequencies of hyperglycemia and hypoglycemia. Each of the eight items is scored on a scale of 0–6.<br>For most questions the higher the score, the greater the satisfaction with treatment. When the perceived frequency of hyperglycemia and hypoglycemia is being addressed, a higher score indicates a problem.	To assess the impact of using the **Accu-Chek Connect** diabetes management system on treatment satisfaction, diabetes distress, and glycemic control in adults with type 1 diabetes and insulin-treated type 2 diabetes. Significant improvements in treatment satisfaction (DTSQc) were observed at 6 months regardless of treatment modality (Multiple daily insulin injections or basal insulin only), diabetes type (T1D or T2D), or HCP practice type (specialist or nonspecialist). **(Mora, 2017)**<br><br>To evaluate the efficacy of **Euglyca application** on patients' glycemic control and satisfaction. Developed for children and adolescents with T1DM.<br>[illegible]
**Diabetes Clinic Satisfaction Questionnaire (DCSQ)**	**1996, Dr Clare Bradley**	Bradley, C. (1994) The Diabetes Treatment Satisfaction Questionnaire: DTSQ. In: Bradley, C., Ed., Handbook of Psychology and Diabetes: A Guide to Psychological Measurement in Diabetes Research and Practice, Harwood Academic Publishers, Chur, 111-132.<br>**(I was not able to download the article)**	To assess patients' satisfaction with diabetes health care delivery	**English**	NA	1	NA	-	-
**Perceptions About Medications for Diabetes (PAM-D)**	**2009 / No information**	Hayes - 2006 - Understanding Diabetes Medications from perspective of Patients TD2	To assess patient's perceptions of medications during the past month	**English**	3	3	NA	-	-
**Satisfaction with Oral Anti-Diabetic Agent Scale (SOADAS)**	**2008 / No information**	Donatti - 2008 - Psychometric evaluation of the Satisfaction with Oral Anti-Diabetic Agent Scale (SOADAS)	The first treatment satisfaction instrument specific to oral anti-diabetic agents (OADs)	**English + 20 more**	None	2	NA	-	-
**Diabetes Medication System Rating Questionnaire-Short Form (DMSRQ-SF)**	**2014 / No information**	Peyrot et al. - 2014 - Research Care Delivery Development and validation of the DMSRQ-Short Form	To provide a comprehensive measure of diabetes medication treatment satisfaction; designed to reduce respondent burden and be short enough for use in clinical practice and research	**English**	1	1	NA	-	-
**Treatment Related Impact Measure for Diabetes (TRIM-D)**	**2008 / Novo Nordisk**	Brod - 2009 - Understanding and assessing the impact of treatment in diabetes - (TRIM-Diabetes and TRIM-Diabetes Device)	To measure treatment related impact on subjects of diabetes medication and **diabetes devices**	**English + many others but check for linguistic validation**	15	Too many results	NA	-	-
**Insulin Treatment Experience Questionnaire (ITEQ)**	**2007 / Sanofi Aventis**	Moock - 2010 - Development and Testing of the Insulin Treatment Experience	To assess subtle but clinically relevant differences in treatment experiences and satisfaction over a wide range of currently available insulin therapy regimens	**German + English**	2	3	NA	-	-
**Insulin Delivery System Rating Questionnaire - IDSRQ**	**2005 / Peyrot M; Rubin RR, (No information for copyright)**	Peyrot - 2004 - Validity and Reliability of an Instrument for Assessi,g Health-related quality of Life and treatment preferences	To assess the impact of different insulin delivery systems.	**English**	5 (could be important, it is about devices)	13	NA	-	-
**Diabetes Medication System Rating Questionnaire (DMSRQ)**<br><br>**Diabetes Medication System Rating Questionnaire short form (DMSRQ-SF)**	**2011 / Mark Peyrot and Richard R. Rubin**<br><br>**2014/ Mark Peyrot and Richard R. Rubin**	Peyrot - 2012 - Validation of a tool to assess medication treatment satisfaction in TD2 - DMSRQ<br><br>Peyrot - 2014 - Development and validation of the Diabetes Medication System Rating Questionnaire-Short Form	To assess satisfaction with any diabetes medication system used by patients with diabetes to control blood glucose (TD2)	**English for USA**	1	4	NA	-	-
**Hypoglycemia Fear Survey or Adult Low Blood Sugar Survey (HFS)**	**1987 - revised 1989 / Instrument copyrighted by the University of Virginia Health System**	Cox - 1987 - Fear of Hypoglycemia - Quantification, Validation and utilization	To measure the degree of fear experience with respect to hypoglycemia	**English + 73 other**	**64** (47, 19)	65	NA	-	-
**Diabetes Fear of Injecting and Self-testing Questionnaire (D-FISQ)**	**1997 / Institute for Research in Extramural Medicine (EMGO Institute)/ Department of Medical Psychology, Faculty of Medicine, Vrije Universiteit, Van der Boechorststraat 7, 1081 BT, Amsterdam, The Netherlands**	Mollema - 2000 - Diabetes Fear of Injecting and Sel-Testing Questionnaire	To quantify the degree of fear of self-injecting insulin and self-testing of blood glucose in adult insulin-treated diabetic patients	**Dutch for the Netherlands + 5 other**	2	14	NA	-	-
**QALY (Quality Adjusted Life Years)**									
**DHP-3D**<br><br>**DHP-5D**	**2017 / No information**	Mulhern et al. - 2017 - Developing preference-based measures for diabetes (DHP-3D and DHP-5D)	Can be used to estimate Quality Adjusted Life Years for use the economic evaluation of diabetes specific interventions.	**English**	NA	1	NA	-	-
**No particular information about it, rarely used.**									
**Diabetes Numeracy Test-15 (DNT-15)**									

DNT- Adolescent and DNT-14 Adolescent
Diabetes Specific Health Literacy Index (DHLI)
Spoken Knowledge in Low Literacy in Diabetes Scale (SKILLD)
Diabetes Tablet Treatment Questionnaire (DTTQ)
Diabetes Food Timing Questionnaire (DFTQ)
Diabetes Health Status Questionnaire (DHS)

PROs (used for diabetes but not specific)	Year / Copyright	Domain or Objective	Languages	Occurrence (based on Clinicaltrial.gov) keys words: Diabetes (TD1,TD2)	Pubmed "PRO full name" diabetes	Pubmed (Occurrences) criteria: "full name PRO" AND Diabetes and ("Mobile application" OR Telemedecine OR telehealth OR "health digital solutions" OR "e-health")	Description (only for the most used PROs based on the number of occurrences of the columns 'F-G-H', taking into account colomn O as well)	References articles (only for the most used PROs based on the number of occurrences of the columns 'F-G-H', taking into account colomn O as well)	PRO mentioned in the guidelines or Used with/for digital solutions
**Adherence**									
**Simplified Medication Adherence Questionnaire (SMAQ)**	**2002 / Hernando Knobel**	To measure adherence to medication	Spanish + English to be checked	7 (NA, 5)	11	NA	NA	-	Fast Track Process for DiGA (specific example)
**Morisky Score**	**2006 / No information**	To measure patients' medication adherence	English + 80	16 (NA, 7)	7	NA	NA	-	Fast Track Process for DiGA (specific example)
**Emotional and social impact**									
**Beck Depression Inventory (BDI)**	**1961 revised 1994 / Aaron T Beck**	To measure the severity of depression in adults and adolescents	English + 73	**67** (12,31)	**457**	1	NA	-	**–**
**Sickness Impact Profile (SIP)**	**1976 / No information**	To measure patients dysfunction through his everyday behavior, and generally related to disease.	English	2 (1,1)	**393**	2	NA	-	**–**
**Affect Balance Scale (ABS)**	**1969 / No copyright**	-	English + 6	6 (3,3)	6	NA	NA	-	**–**
**HADS (Hospital Anxiety Depression Scale)**	**1960/ nferNelson Publishing Company Ltd permissions@nfer-nelson.co.uk**	To detect states of anxiety and depression	English + 75	**66** (8,24)	19	NA	NA	-	**–**
**Symptom Checklist SCL-90**	**1975 / Derogatis LR**	Designed to screen for a broad range of psychological problems and symptoms of psychopathology, it is also useful as a progress or outcomes measurement instrument	English + 10	1 (NA, 1)	5	NA	NA	-	Fast Track Process for DiGA (specific example)
**PSDI (Positive Symptom Distress Index)**	**Not found**	To measure the distress level	English	NA	5	NA	NA	-	Fast Track Process for DiGA (specific example)
**PST (Positive Symptom Total)**	**Not found**	-	English	8 (2, 4)	2	NA	NA	-	Fast Track Process for DiGA (specific example)
**Quality of life**									
**EQ-5D**	**1990 / The Euroqol Group**	To assess health outcome from a wide variety of interventions on a common scale, for purposes of evaluation, allocation and monitoring	English + 181	**255** (33, 124)	**580**	3	The EQ-5D assesses five domains of generic health related QoL (mobility, self care, usual activities, pain and discomfort, anxiety and depression) and can generate either a health state (of 243 different states) or a single summary score (higher scores reflect better health related QoL). The EQ-5D has shown good validity and responsiveness and has been recommended for patients with diabetes and, more cautiously, for patients with chronic obstructive pulmonary disease and heart failure. It is a generic measure of health status that provides a simple descriptive profile and a single index value that can be used in the clinical and economic evaluation of health care and in population health surveys	Study of the effects of the group-based PRoactive Interdisciplinary Self-MAnagement (PRISMA) training program on self-reported and clinical outcomes in patients with type 2 diabetes treated in general practice. No significant results (**Du Pon, 2019**)<br><br>Assess the effect of second generation, home based telehealth on health related quality of life, anxiety, and depressive symptoms over 12 months in patients with long term conditions. No significant results (**Cartwright, 2013**)<br><br>Assess the feasibility of using a photographic foot imaging device (PFID) as a tele-monitoring tool in the home environment of patients with diabetes who were at high risk of ulceration. Health-related quality of life (EQ-5D visual analogue scale), increased from 7.5 at [illegible]	Principles of evaluation by the CNEDIMTS for medical devices for QoL<br><br>CDRH PRO Compendium (specific example, FDA)
**SF-36**	**1990 / The Medical Outcomes Trust (MOT) Dr. J. Ware**	To measure generic health concepts relevant across age, disease, and treatment groups	English + 160	**291**(32,142)	**1113**	7	A 36-item short-form (SF-36) was constructed to survey health status in the Medical Outcomes Study. The SF-36 was designed for use in clinical practice and research, health policy evaluations, and general population surveys. The SF-36 includes one multi-item scale that assesses eight health concepts: 1) limitations in physical activities because of health problems; 2) limitations in social activities because of physical or emotional problems; 3) limitations in usual role activities because of physical health problems; 4) bodily pain; 5) general mental health (psychological distress and well-being); 6) limitations in usual role activities because of emotional problems; 7) vitality (energy and fatigue); and 8) general health perceptions. The survey was constructed for self-administration by persons 14 years of age and older, and for administration by a trained [illegible]	The objective of this study was to test whether the use of a mobile phone-based self-management system used for 1 year, with or without telephone health counseling by a diabetes specialist nurse for the first 4 months, could improve glycated hemoglobin A1c (HbA1c) level, self-management, and health-related quality of life compared with usual care. No significant results with health-related quality of life (SF-36). (**Holmen, 2014**)<br><br>This study examined the effects on QOL-relevant psychosocial measures of a widely available commercial weight loss program enhanced for individuals with T2DM. No significant treatment effect on SF-36 scores. (**Holland-Carter, 2017**)<br>Evaluate the effect of a telemedicine system on maternal and fetal outcome in women with diabetes. For QoL (SF-36), women in the [illegible]	Principles of evaluation by the CNEDIMTS for medical devices for QoL<br><br>Fast Track Process for DiGA (specific example)<br><br>CDRH PRO Compendium (specific example, FDA)
**SF-20**	**NA**	To measure generic health concepts relevant across age, disease, and treatment groups	English	5(1,3)	49	NA	NA	-	**–**
**Well-Being Questionnaire (WBQ) - 12 items & 28 items**	**2000 / Prof C. Bradley, Royal Holloway University of London Email: c.bradley@rhul.ac.uk**	QoL	English	2 (1,1)	NA	NA	NA	-	**–**
**Symptoms and severity**									
**Numerating Rating Scale (NRS)**	**1984 / No information**	To measure pain	English	**86** (NA, 17)	NA	NA	NA	-	Fast Track Process for DiGA (specific example)
**GSI (Global Severity Index)**	**Not found**	To help quantify a patient's severity-of-illness	English	6 (NA, 4)	10	NA	NA	-	Fast Track Process for DiGA (specific example)
**Empowerment & health literacy**									
**HLS-EU-Q**	**2013 / Creative Commons License**	To measure health literacy	English	3 (NA, 2)	2	NA	NA	-	Fast Track Process for DiGA (specific example)

N°Items	Format	Time administration	Questionnaire (.pdf downloaded)	PRO mentioned in the guidelines or Used with/for digital solutions	Pubmed - Correlation HbA1c (Occurrences) criteria: "diabetes" (diabetes type 1 / diabetes type 2) "full name PRO" "HbA1c" filter: Full text	Pubmed - Correlation HbA1c<br>PROs has been used to find a correlation with Hb1Ac ? (Yes/No, if yes what results?)	Pubmed - Correlation HbA1c<br>Here, only abstracts where medical devices are used have been reported
**17-19**	Rating from 1 (haven't done it at all) to 5 (have always done it).	< 10 minutes	**Yes**	**–**			
14 multiple choice questions administered verbally by healthcare professionals	Items 1-10 are scored as follows: 1= none of the time, 2 = some of the time, 3 = most of the time, and 4 = all of the time. Item 11 is reversed scored. The ARMS-D total is a sum of all items for a possible score ranging from 11-44. The ARMS-D refill sub-scale is a sum of 4 items for a possible score ranging from 4-16. The ARMS-D medication taking sub-scale is a sum of 7 items for a possible score ranging from 7-28. Lower scores indicate better adherence.	NA	**No**	**–**			
**11**	Scores from 0 to 7	< 10 minutes	**Yes**	**–**	46 (10 TD1, 40 TD2)	The PRO has been used to explore the correlation with HbA1c in ***10 studies.***<br>*When precised a negative correlation was found.*<br><br>*A list of these papers is reported:*<br>Aga et al. - 2020 - Relationships of diabetes self-care behaviours to glycaemic control in adults with type 2 diabetes and comorbid heart failure<br>Ausili et al. - 2017 - Self-care, quality of life and clinical outcomes of type 2 diabetes patients: an observational cross-sectional study<br>Ali H AlQahtani et al. - 2020 - Levels of Practice and Determinants of Diabetes Self-Care in	None
**25**	NA	NA	**No**	**–**			
**[UK] 20 - [IT] 15**	Since DMSES consists of two factors, two scores are necessary. Score 'Disease Management' is the weighted mean of items 1, 2, 3, 6, 14, 15. Score 'Lifestyle Management' is the weighted mean of items 4, 5, 7, 8, 9, 10, 11, 12, 13. Both of them range from 0 to 10: 0–3 denotes low levels of self-efficacy, 4–6 intermediate levels of selfefficacy, 7–10 high levels of self-efficacy	10-20 minutes	**No**	**–**			
**4**	a seven-point Likert scale indicating level of agreement (1–7, ranging from 'not at all true' to 'very true'); higher scores represent better respondent's performance.	< 5 minuutes	**No**	**–**			
**16 item in first version.<br>4 subscales in total (20 + 7) items**	NA	NA	**Yes**	**–**			
7	likert scale (1-5), total range (1-35) where lower scores indicate better QoL	NA	**No**	**–**			
**17**	6-point Likert Scale questions where 1 indicates no problem and 6 indicates a very serious problem.	10 minutes	**No**	**Used for the reimbursement of Freestyle Libre**	61 (25 TD1, 38 TD2)	The PRO has been used to explore the correlation with HbA1c in 28 studies.<br>Most of the time a positive correlation was found.<br><br>*A list of these papers is reported:*<br><br>Fisher et al. - 2012 - When is diabetes distress clinically meaningful?: establishing cut points for the Diabetes Distress Scale. Diabetes Care.<br>Van Bang Nguyen et al. - 2020 - Diabetes-Related Distress and Its Associated Factors Among Patients with Diabetes in Vietnam	Khan et al. - 2018 - Investigating the Association Between Diabetes Distress and Self-Management Behaviors: Insulin pomps<br>Tyndall et al. - 2019 - Marked improvement in HbA1c following commencement of flash glucose monitoring in people with type 1 diabetes: Flash Glucose Monitor
**3 versions:<br>20-item version (original)<br>5-item version<br>1-item version**	Each question has five possible answers with a value from 0 to 4, with 0 representing "no problem" and 4 "a serious problem".	NA	**Yes**	**Comprised in the COMET COS which is mentioned in the Evidence standards framework for DHT for tier 3b (NICE)**	45 (21 TD1, 29 TD2)	The PRO has been used to explore the correlation with HbA1c in ***15 studies.***<br>Most of the time a positive correlation was found.<br><br>*A list of these papers is reported:*<br>Evans et al. - 2019 - Psychometric Properties of the Parent and Child Problem Areas in Diabetes Measures.<br>Chin et al. - 2017 - The validity and reliability of the English version of the diabetes distress scale for type 2 diabetes patients in Malaysia.<br>Hsu et al. - 2013 - Developing and psychometric testing of a short-form problem areas in	None
**32<br>18**	NA	NA	**No**	**Used for the reimbursement of Diabeo (DHP type not specified)**			
**2**	Rating from 1-6 where 1 is "not problem" and 6 is "A very serious problem"<br><br>DDS-17 is administered to those who screen positive with DDS-2	NA	**Np**	**–**			
**19**	Strongly disagree<br>Disagree<br>Unsure<br>Agree<br>Strongly Agree	< 10 minutes	**Yes**				
34 items, 8 domains (psychology-fatigue, psychology-cognitive, neurologypain, neurology-sensory, cardiology, ophthalmology, hypoglycemia, and hyperglycemia)	Simptoms yes no, if no 0 score, if yes give a score from 1 to five	NA	**No**	**–**			
DCP scores differed by diabetes type and treatment.<br>In types 1 and 2 patients combined, correlations with HbA1c were noted for three DCP scales—control<br>problems, self-care ability, and self-care adherence.<br>For type 2 patients not using insulin, three additional scales correlated with HbA1c<br>, i.e., social and personal factors, negative attitude, and medical barriers	NA	NA	**Yes**	**–**			

**20**	Never true for me (0), Sometimes true for me (1) Often true for me (2), Always true for me (3)	10 minutes	**Yes**	–			
**16**	NA	10 minutes	**No**	–			
**46**	5-point Likert scale. 1 - Very Satisfied, 5 - very dissatisfied.<br><br>three main domains, namely, "satisfaction," "impact," and "worry."	NA	**Yes**	**Principles of evaluation by the CNEDIMTS for medical devices**<br><br>**Used for the reimbursement of Freestyle Libre**<br>**Used for the reimbursement of Diabeo**	76 (53 TD1, 19 TD2)	The PRO has been used to explore the correlation with HbA1c in ***31 studies.***<br>Most of the time a negative correlation was found.<br><br>*A list of these papers is reported:*<br>Costa et al. - 2015 - Quality of life of adolescents with type 1 diabetes.<br>Martins et al. – 2018 - Health-related quality of life in a cohort of youths with type 1 diabetes.<br>Hoey et al. – 2017 – Hvidoere Smiley Faces: International diabetes quality of life assessment tool for young children.<br>Bujang et al. – 2017 - Validation of the Malay version of Diabetes Quality of Life (DQOL)	Wikby et al. – 1998 - Metabolic control, quality of life, and negative life events: a longitudinal study of well-controlled and poorly regulated patients with type 1 diabetes after changeover to insulin pen treatment: Insulin pen
**?**	NA		**No**	–			
**19**	NA	NA	**No**	–			
**39**	5 dimension:<br>- L'énergie et la mobilité : 15 items.<br>- Le contrôle du diabète : 12 items.<br>- Les limites sociales : 5 items.<br>- L'inquiétude et l'anxiété : 4 items.<br>5. L'activité sexuelle : 3 items	NA	**No**	–			
**?**	NA	NA	**No**	–			
**long and short verion (-R)**	NA	NA	**No**	–			
?	NA	NA	**No**	–			
28, short version of 8 items	with response categories of 'strongly disagree', 'somewhat disagree', 'neutral', 'somewhat agree', and 'strongly agree'.<br><br>An overall score for the DES is calculated by summing all of the item scores and dividing by 8 for a possible score range of 1-5 where a higher score indicates a higher level of empowerment.	NA	**Yes**	–	21 (6 TD1, 17 TD2)	The PRO has been used to explore the correlation with HbA1c in ***10 studies.***<br>Most of the time a negative correlation was found.<br><br>*A list of these papers is reported:*<br>Silveira et al. - 2019 - Low empowerment and diabetes regimen distress are related to HbA1c in low income type 1 diabetes patients in a Brazilian tertiary public hospital<br>Łuczyński et al. - 2019 - The Empowerment of Adolescents with Type 1 Diabetes Is Associated with Their Executive Functions<br>Thew Hui Zhu et al. - 2019 - Diabetes empowerment scores among type 2 diabetes mellitus	None
60	NA	NA	**yes**	–			
**NA**	Rating on a scale from 1-10 where 1 is not at all confident and 10 is totally confident	NA	**No**	–			
**8**	7 point scale<br><br>scale ranging from zero (e.g., "very dissatisfied","very inconvenient") to six (e.g., "very satisfied", "very convenient") for the first factor.<br><br>Scale ranging from zero being "none of the time" to six being "most of the time" for the second factor.<br><br>The questionnaire is composed of two different factors. The first factor assesses treatment satisfaction and the second one the burden from hyper-and hypoglycemia	NA	**Yes**	**Principles of evaluation by the CNEDIMTS for medical devices**<br><br>**Used for the reimbursement of Freestyle Libre**	99 (37 TD1, 66 TD2)	The PRO has been used to explore the correlation with HbA1c in **14 studies.**<br>Most of the time a negative correlation was found.<br><br>*A list of these papers is reported:*<br>Haas et al. - 2020 - Treatment satisfaction correlated with glycaemic control and burden of diabetes in Swedish adolescents with type 1 diabetes.<br>Hayashi et al. - 2018 - Factors associated with treatment satisfaction in patients with type 2 diabetes mellitus using oral glucose-lowering agents: a cross-sectional study in urban districts in Japan	Chatzakis et al. - 2019 - The Beneficial Effect of the Mobile Application Euglyca in Children and Adolescents with Type 1 Diabetes Mellitus: A Randomized Controlled Trial: mobile app Euglyca
**NA**	NA	NA	**No**	–			
**37**	A four-category how often response scale was used for all items:<br>"None of the time," "Some of the time," "Quite a bit of the time," and "All of the time."	longer	**No**	–			
**?**	NA	NA	**No**	–			
**54 (long version)**<br><br>**20 (short version, used in online study)**	NA	NA	**No**	–			
**?**	NA	NA	**No**	–			
?	NA	NA	**Yes**	–			
The IDSRQ has seven sub-scales (satisfaction, interference of treatment with daily activities, diabetes-related worries, clinical efficacy, psychological well-being, social burden, and treatment preference). Each sub-scale is calculated as a mean of their corresponding items with scores ranging from 0-100. Higher scores represent higher levels of satisfaction, perceived clinical efficacy, and psychological well-being, interference with daily activities, diabetes-related worries, and social burden.	A standardized questionnaire including several questions with ordinal response scales from 1 to 4 or 1 to 5.	NA	**No**	–			
?	Rating from 0-3 where 0=Does not apply to me, 3= Applies to me very much	NA	**No**	–			
				–			
?	NA	NA	**No**	–			
**?**	NA	NA	**No**	–			
