# Supplementary material for: Evaluating Digital Health Solutions in Diabetes and the Role of Patient-Reported Outcomes: Targeted Literature Review
Source: JMIR Diabetes. 2025 Jun 4;10:e52909. doi: 10.2196/52909 (PMC12158397; doi:10.2196/52909)
Supplement: Multimedia Appendix 2 [file diabetes-v10-e52909-s002.docx]

**Supplementary material 4: Search strategy per database**

| **Database** | **Search strategy and search keywords** |
| --- | --- |
| PubMed | 1. “full name of PROM” AND diabetes 2. “full name of PROM” AND “type 1 diabetes” 3. “full name of PROM” AND “type 2 diabetes” 4. (“full name of PROM” AND diabetes) AND (“mobile application” OR telemedicine OR telehealth OR “health digital solutions” OR “e-health”)   “full name of PROM”:   - “Summary of Diabetes Self-Care Activities” - “Diabetes Distress Scale” - “Problem Areas in Diabetes” - “Diabetes Empowerment Scale” - “Diabetes Quality of Life” - “Diabetes Treatment Satisfaction Questionnaire” - “Beck Depression Inventory” - “Sickness Impact Profile” - “EQ-5D” - “SF-36” |
| ClinicalTrials.gov | 1. “full name of PROM” AND diabetes 2. “full name of PROM” AND “type 1 diabetes” 3. “full name of PROM” AND “type 2 diabetes”   “full name of PROM”:   - “Summary of Diabetes Self-Care Activities” - “Diabetes Distress Scale” - “Problem Areas in Diabetes” - “Diabetes Empowerment Scale” - “Diabetes Quality of Life” - “Diabetes Treatment Satisfaction Questionnaire” - “Beck Depression Inventory” - “Sickness Impact Profile” - “EQ-5D” - “SF-36” |
