# Supplementary material for: Evaluating Digital Health Solutions in Diabetes and the Role of Patient-Reported Outcomes: Targeted Literature Review
Source: JMIR Diabetes. 2025 Jun 4;10:e52909. doi: 10.2196/52909 (PMC12158397; doi:10.2196/52909)
Supplement: Multimedia Appendix 3 [file diabetes-v10-e52909-s003.pdf]

	PROMs listed in the literature review or mentionned by the board	HTA Guidelines	Feedback from the advisory board
Domains	*PROMs*	*Importance in the guidelines*	*Comment of the adboard*
Quality of Life	WHO 5<br>EQ5D<br>SF-36<br>SF-20<br>WBQ<br>DQOL<br>ADDQOL<br>DSQOLS<br>ADDQOL<br>D-39<br>DQLCTQ<br>PedsQL™<br>DIMS	+++ (NICE, BfArM)<br>+ HAS	*About the domain:* QoL still remains a most important PRO to assess.<br><br>*About the PROMs:*<br><br>General comment: Generally, the use of a PROM depends if it is for clinical use or to answer scientific questions.<br><br>EQ5D is used frequently because it is directly linked to the reimbursment processes (QALY) in many countries.
Adherence	MAQ<br>ADQ-I<br>ADQ-C<br>ARMS-D<br>Morisky Score	++ (BfArM)	*About the domain:* Adherence to treatment and with DHS is not the same.<br><br>*About the PROMs:* They are not necessarily needed with DHS. This domain can be evaluated through direct measure in the DHS.
Health Literacy	LAD<br>SED-D<br>HLS-EU-Q	+ (BfArM)	*About the domain:* It is rarely measured, but interesting when proving the value of a DHS.<br>*About the PROMs:* They are not meeting the expectations in assessing the patient outcome.
Acceptability	DTSQ	++ (NICE & HAS)	-
Patient empowerment	DES	++ (NICE)	-
Symptom severity	NRS<br>GSI	+++ (BfArM)<br>+ (NICE)	-
Coping with illness-related difficulties	There could be an overlap with some dimensions of the SDSCA (domain: self-management) but this is not the primary goal of the PROM.	+ (BfArM)	-

Reduction of therapy-related efforts and strains (for patients and their relatives)	-	+ (BfArM)	-
Enhanced safety	-	+ (BfArM)	-
User satisfaction	DTSQ (can be adapted)	+ (NICE & BfArM)	-
Engagement	-	+ (NICE)	*About the PROMs: They are not necessarily needed with DHS. This domain can be evaluated through direct measure in the DHS*
Autonomy	-	+ (NICE & HAS & BfArM)	-
Additional Domains or PROMs - The domains may overlap with some of the above			
Emotional and social impact	PHQ5 or 8<br>BDI<br>SIP<br>HADS<br>ABS<br>SCL-90<br>PSDI<br>PST<br>DDS<br>PAID<br>DHP-1<br>DHP-18 (eDHP-18)<br>DDS2<br>DSAS-2<br>DSC-R<br>DSC-2<br>DCP	-	*About the PROMs:* PHQ5 or 8 (used for depression), PAID were underlined by the board
Self-management	SDSCA<br>DSMP<br>DMSES<br>PCDS<br>DSMQ<br>DSMQ-R<br>ADS	-	*About the domain:* Self-care behaviour is a critical outcome to address and the available technologies are often not adapted to measure it.<br>*About the PROMs:* SDSCA is frequently used, but has been validated some decades ago and may therefore not be useful for DHS.
Diabetes acceptance	DAS<br>DMSAT	-	-

Patient treatment satisfaction	DTSQ / DTSQs<br>DCSQ<br>PAM-D<br>SOADAS<br>DMSRQ-SF<br>TRIM-D<br>IDSRQ<br>DMSRQ<br>DMSRQ-SF<br>HFS<br>HAS<br>D-FISQ	-	*About the PROMs:* DTSQ is not so relevant here, HFS-II is really important and frequently used, was missing from the list of PROMs, there is also HAS as a new tool that was validated.
Disease Knowledge	-	-	What the patients have to know to manage their diabetes is not explored yet or covered by existing PROMs: it is useful for patients to have better knowledge about their disease but it is currently not seen as an endpoint (in a reimbursement process, as long as there is no link to a better QoL or management of HbA1c). It can be considered as a step in the journey of the patient to shift the patients' attitude.
E-health literacy	-	-	Using DHS could help increase patients' e-health literacy: trainings and a good software design could help address this issue through items like error prevention, minimalistic design, error-free and simple use of a system, etc.
Sexual life	Measured in QoL PROMs (not as single outcomes)	-	Mentioned by the adboard, no adapted PROMS
Family life	Measured in QoL PROMs (not as single outcomes)	-	Mentioned by the adboard, no adapted PROMS
Well-being at work	Measured in QoL PROMs (not as single outcomes)	-	Mentioned by the adboard, no adapted PROMS
Digital burden: Evaluate problems with DHS like data overload, fear of being monitored, side effects of digital use	-	-	Mentioned by the adboard, no adapted PROMS

Additional comment: EMA is a digital instrument, it was mentioned as an interesting methodology by the advisers when looking at digital ways to measure PROM
